# A comparative analysis of survival outcomes and adverse effects between preoperative brachytherapy with radical surgery and concurrent chemoradiotherapy in patients with locally advanced cervical cancer

**DOI:** 10.3389/fonc.2025.1511748

**Published:** 2025-02-28

**Authors:** Yuna Niu, Chengchao Du, Yeqin Zhou, Miao Zhang, Qi Guo, Honggui Zhou

**Affiliations:** ^1^ Department of Gynecology and Obstetrics, Affiliated Hospital of North Sichuan Medical College, Nanchong, China; ^2^ North Sichuan Medical College, Nanchong, China; ^3^ North Sichuan Medical College Innovation Centre for Science and Technology, Nanchong, China; ^4^ Department of Oncology, Affiliated Hospital of North Sichuan Medical College, Nanchong, China

**Keywords:** locally advanced cervical cancer, concurrent chemoradiotherapy, preoperative brachytherapy, propensity score matching, progression-free survival

## Abstract

**Background:**

To compare the long-term efficacy and adverse effects of preoperative brachytherapy combined with radical surgery versus concurrent chemoradiotherapy (CCRT) in patients with locally advanced cervical cancer (LACC).

**Methods:**

This retrospective study analyzed 161 patients with LACC treated at the Affiliated Hospital of North Sichuan Medical College between January 2015 and December 2020. Of these, 76 patients underwent preoperative brachytherapy combined with radical surgery (study group), while 85 received CCRT (control group). After propensity score matching (PSM) to minimize confounding, 124 patients (62 per group) were included in the analysis. Survival outcomes and prognostic factors were evaluated using Kaplan-Meier survival analysis and Cox regression models. Adverse effects of treatment were compared between the groups.

**Results:**

After PSM, the 5-year progression-free survival (PFS) rate in the study group was significantly higher than that in the control group (81.2% vs. 62.7%, *P*<0.05). There was no significant between-group difference regarding the 5-year overall survival (OS) rate (81.4% vs. 74.9%, *P*=0.41). Multivariate analysis identified treatment modality (preoperative brachytherapy combined with radical surgery vs. CCRT) as an independent prognostic factor for PFS (HR: 0.458, 95% CI 0.221–0.945, *P*=0.035). The study group had significantly lower rates of grade 2 acute radiation enteritis, grade 3-4 leukopenia, and anemia compared to the control group (*P*<0.05), with no significant differences observed in other adverse effects (*P*>0.05).

**Conclusion:**

Preoperative brachytherapy combined with radical surgery may help improve the PFS of patients with LACC, with fewer adverse effects, making it a potentially viable treatment option for these patients.

## Introduction

Cervical cancer is the second most common malignancy among women in developing countries, surpassed only by breast cancer (1). Over 50% of cervical cancer cases are diagnosed at an advanced stage (2), with locally advanced cervical cancer (LACC) being particularly aggressive, characterized by high rates of lymphatic metastasis and poor local control. The National Comprehensive Cancer Network (NCCN) clinical practice guidelines recommend platinum-based concurrent chemoradiotherapy (CCRT) as the standard treatment for ≥ stage IIB LACC (3). Despite this treatment approach, the 5-year recurrence rate remains high, at approximately 40–50%, and nearly 30% of patients succumb to the disease within 5 years (4). Moreover, CCRT is associated with considerable treatment-related toxicity, including hematological, gastrointestinal, and genitourinary complications, with up to 35% of patients experiencing severe late-stage toxicity within 3 years post-treatment (5). Thus, exploring therapeutic strategies that offer improved efficacy with reduced toxicity is a key research imperative. Recent studies suggest that preoperative radiotherapy may reduce tumor burden, decrease tumor cell viability, enhance surgical resection rates, and reduce local recurrence in patients with LACC (6, 7). However, there is a paucity of data on the long-term survival and quality of life following preoperative brachytherapy combined with radical surgery in these patients. In the present study, we retrospectively analyzed the clinical data of 124 patients with LACC to compare survival outcomes and adverse effects between preoperative brachytherapy combined with radical surgery and CCRT, after propensity score matching (PSM) (8, 9) to control for baseline confounding factors.

## Materials and methods

### Study population

Clinical data pertaining to 161 patients with stage IB3 to IIIC1 [2018 FIGO staging (10)] cervical cancer treated at the Affiliated Hospital of North Sichuan Medical College between January 2015 and December 2020 were retrospectively analyzed. Patients were categorized into two groups based on the treatment regimen: the study group (preoperative brachytherapy combined with radical surgery, n=76) and the control group (CCRT, n=85). The study was approved by the Ethics Committee of the Affiliated Hospital of North Sichuan Medical College (approval number: 2024ER496–1).

The inclusion criteria were as follows: 1) primary cervical cancer confirmed by pre-treatment biopsy; 2) stages IB3 to IIIC1; 3) ECOG performance status score ≤ 2. The exclusion criteria were: 1) prior antitumor therapy; 2) concomitant significant organ diseases or primary tumors at other sites; 3) previous radiotherapy at other sites; 4) additional postoperative adjuvant chemotherapy, radiotherapy, or chemoradiotherapy for high-risk factors based on pathology (11); 5) lack of CT or MRI assessment of tumor and lymph node status; 6) allergy to chemotherapeutic agents; 7) incomplete medical records.

### Treatment methods

#### Study group

Prior to surgery, patients received preoperative treatment consisting of three-dimensional image-guided afterloading intracavitary brachytherapy. The treatment plan was developed by a multidisciplinary team, delivering a dose of 6 Gy/Fx (12, 13) to 90% of the clinical target volume (CTV), administered 3–4 times (13) before surgery. Following brachytherapy, patients underwent a minimum 2-week rest period, after which they underwent gynecological and imaging assessments to evaluate tumor response and reduction. If no contraindications were identified, patients underwent abdominal radical hysterectomy [Querleu-Morrow classification-C type (14)], bilateral salpingo-oophorectomy, and pelvic lymphadenectomy.

#### Control group

Patients underwent radical pelvic radiotherapy (PRT), targeting the region from the bifurcation of the abdominal aorta to the lower edge of the obturator, including the entire vagina for stage IIIA disease. In cases requiring extended coverage, extended-field radiotherapy (EFRT) was employed, expanding PRT coverage to the level of the renal arteries or the first lumbar vertebra. The PRT dose was 45–50.4 Gy/25–28Fx, delivered 5 times per week. An additional 5–10 Gy was administered for parametrial and/or pelvic wall invasion, while patients with positive pelvic lymph nodes received an additional 10–20 Gy. Following external irradiation, patients received 4–5 sessions of high-dose-rate three-dimensional intracavitary afterloading treatment, delivering a single dose of 6 Gy/Fx to 90% CTV, 1–2 times per week. Concurrent chemotherapy with platinum-based drugs (cisplatin 20–30 mg/m²) was administered weekly for at least 4 cycles.

### Treatment details

Study Group: Out of 62 patients, 10 patients (16.13%) received 3 sessions of high-dose-rate (HDR) intracavitary brachytherapy, while 52 patients (83.87%) received 4 sessions. The average preoperative brachytherapy dose in the entire group was 23.03 ± 2.22 Gy. The tumor diameter after preoperative brachytherapy in the study group was 20.02 ± 5.88 mm.

Control Group: The average radiotherapy doses administered in the control group were as follows: planning tumor volume (PTV): 48.23 ± 2.06 Gy; Paracervical clinical target volume P-CTVp: 56.48 ± 3.50 Gy; and Enlarged Pelvic Lymph Nodes P-GTVnd: 57.26 ± 5.01 Gy. Of the 62 patients, 15 (24.19%) received 4 sessions of high-dose-rate (HDR) intracavitary brachytherapy, while 47 (75.81%) received 5 sessions. The average dose of HDR intracavitary brachytherapy for the entire group was 28.55 ± 2.59 Gy. Chemotherapy for the control group consisted solely of cisplatin. The average single dose of cisplatin was 45.69 ± 3.60 mg while the mean total dose was 227.91 ± 20.25 mg. Sixty-one patients (98.4%) received five chemotherapy sessions, while one patient (aged 73 years) received 4 chemotherapy sessions. Among the 62 patients, 60 (96.78%) completed the entire treatment course within 8 weeks, while 2 (3.23%) completed the treatment within 10 weeks. Additional details of treatment in the control group are presented in **Supplementary Material**.

### Observation indicators

#### Clinical efficacy

The primary endpoint was progression-free survival (PFS), defined as the time from the start of treatment to the first occurrence of disease progression or death. The secondary endpoint was overall survival (OS), defined as the time from the start of treatment to death or the last follow-up date.

#### Assessment of adverse effects

Adverse effects were recorded from the start of radiotherapy up to 3 months, with the most severe adverse effects noted. Chemoradiotherapy adverse effects were evaluated using the Common Terminology Criteria for Adverse Events (CTCAE) version 5.0 (15), focusing on gastrointestinal, genitourinary, and hematological reactions.

#### Follow-up

Patients were monitored through regular outpatient visits or telephonic follow-ups, with the last follow-up date set on January 31, 2024, or the date of death, whichever occurred first.

### Statistical analysis

Data were analyzed using IBM SPSS (26.0), R (4.2.2), and RStudio (2022.07.2 + 576). PSM (8, 9) was performed using SPSS 26.0, with logistic regression employed to estimate propensity scores, followed by 1:1 nearest-neighbor matching with a caliper size of 0.03 (16, 17). The adjusted variables included age, pathological type, FIGO stage, primary tumor diameter, and pelvic lymph node metastasis. Normally distributed continuous variables were expressed as mean ± standard deviation and analyzed using the independent *t*-test; non-normally distributed continuous variables were expressed as median (P25, P75) and analyzed using the non-parametric Wilcoxon signed-rank test. Categorical variables were expressed as frequency (percentage) and analyzed using Pearson chi-square or Fisher’s exact tests. Kaplan-Meier survival curves were generated using the “survminer” package in RStudio and between-group differences in survival outcomes were assessed using the log-rank test. Cox proportional hazard models were used for multivariate analysis. *P*-values < 0.05 were considered indicative of statistical significance.

## Results

### General patient characteristics

The average age of 124 patients in the PSM cohort was 52.00 (47.00, 56.00) years, with no significant difference between the study group (52.00 [47.00, 58.25] years) and the control group (52.00 [48.50, 55.25] years). The follow-up period ended on January 31, 2024, with a median follow-up time of 52.50 months. The other clinical characteristics are summarized in **Table 1**.

**Table 1:** Clinical characteristics of the study population.

Characteristic	Before PSM (*n*=161)			After PSM (*n*=124)		
	Study group (*n*=76)	Control group (*n*=85)	*P* value	Study group (*n*=62)	Control group (*n*=62)	*P* value
Age (years), M (P25, P75)	48.50 (46.00, 55.75)	53.00 (49.00, 60.50)	0.005*^a^	52.00 (47.00, 58.25)	52.00 (48.50, 55.25)	0.669^a^
Histology, n (%)						
SCC	71 (93.4)	80 (94.1)	1.000^b^	59 (95.2)	58 (93.5)	1.000^b^
Non-SCC	5 (6.6)	5 (5.9)		3 (4.8)	4 (6.5)	
FIGO Stage, n (%)						
IB3-IIB	59 (77.6)	62 (72.9)	0.492^c^	50 (80.6)	52 (83.9)	0.638^c^
IIIA-IIIC1	17 (22.4)	23 (27.1)		12 (19.4)	10 (16.1)	
Primary tum-or size (mm)	40.38 ± 12.27	43.73 ± 12.52	0.089^d^	42.15 ± 11.80	41.90 ± 11.95	0.910^d^
Pelvic MLNs, n (%)						
No	59 (77.6)	74 (87.1)	0.115^c^	50 (80.6)	52 (83.9)	0.638^c^
Yes	17 (22.4)	11 (12.9)		12 (19.4)	10 (16.1)	

^a^ Wilcoxon Rank-Sum test; ^b^ Fisher’s Exact test; ^c^ Chi-Square test; ^d^ Independent Samples *t*-test; *P<0.05.

PSM, propensity score matching; FIGO, International Federation of Gynecology and Obstetrics; SCC, squamous cell carcinoma; MLNs, metastatic lymph nodes.

### Survival outcomes

**Figure 1:**
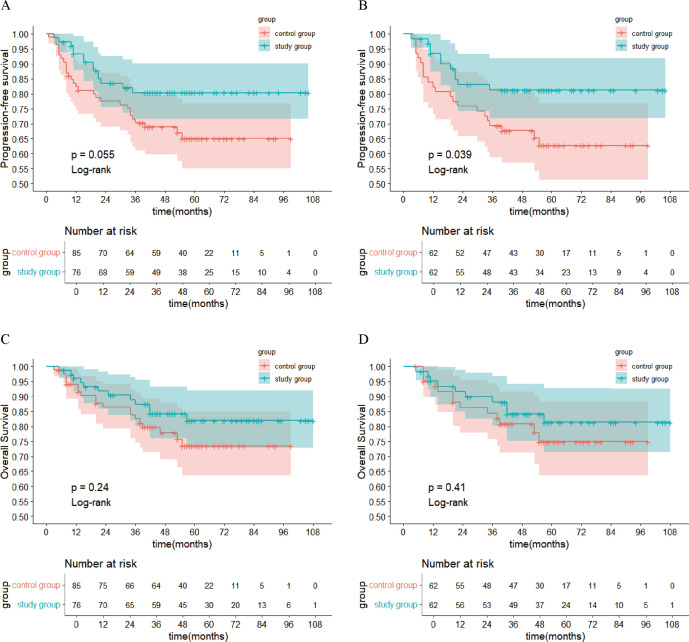
Kaplan-Meier Survival Curves. **(A)** PFS in each group before PSM; **(B)** PFS in each group after PSM; **(C)** OS in each group before PSM; **(D)** OS in each group after PSM. PFS, progression-free survival; PSM, propensity score matching; OS, overall survival.

After PSM, the 1-year, 3-year, and 5-year PFS rates in the study group (88.3%, 81.2%, and 81.2%, respectively) were significantly higher than those in the control group (80.6%, 65.2%, and 62.7%, respectively; *P*<0.05). In the study group, 11 patients experienced recurrence (2 cases of local recurrence, 4 cases of locoregional recurrence, and 5 cases of distant metastasis). In the control group, 22 patients experienced recurrence. In the study group, 10 patients died, while in the control group, 13 patients died (The OR values are provided in the **Supplementary Material**). The survival curves for both groups are presented in **Figure 1**.

### Prognostic factors

Age, pathological type, FIGO stage, primary tumor size, pelvic lymph node metastasis, and treatment modality were included in univariate analysis. The results indicated that treatment modality and primary tumor diameter were associated with PFS (*P*<0.1). On multivariate analysis, preoperative brachytherapy combined with radical surgery was an independent prognostic factor for PFS (hazard ratio [HR]: 0.458, 95% confidence interval [CI] 0.221–0.945, *P*=0.035), reducing the risk of disease progression by 54.2% (0.458 times) compared to CCRT (**Table 2**).

**Table 2:** Results of univariate and multivariate Cox regression analysis for PFS in the PSM cohort.

	Univariate Cox			Multivariate Cox		
	*HR*	95% *CI*	*P* value	*HR*	95% *CI*	*P* value
Age (years)	0.963	0.915–1.013	0.147	0.967	0.916–1.020	0.219
Histology (Non-SCC vs. SCC^#^)	1.573	0.479–5.160	0.455	1.768	0.532–5.879	0.353
FIGO Stage (IIIA-IIIC1 vs. IB3-IIB^#^)	1.436	0.623–3.312	0.197	1.254	0.527–2.982	0.609
Primary tumor size (mm)	1.027	0.996–1.059	0.086*	1.020	0.986–1.054	0.261
Pelvic MLNs (Yes vs. No^#^)	1.436	0.623–3.312	0.395	–	–	–
Treatment Method (Preoperative brachytherapy Combined with Surgery vs CCRT^#^)	0.475	0.230–0.979	0.044**	0.458	0.221–0.945	0.035**

^#^Control Group; *P<0.1, **P<0.05.

PFS, progression-free survival; PSM, propensity score matching; CI, confidence interval; SCC, squamous cell carcinoma; MLNs, metastatic lymph nodes; FIGO, International Federation of Gynecology and Obstetrics; CCRT, concurrent chemoradiotherapy.

### Subgroup analysis and interaction analysis

**Figure 2:**
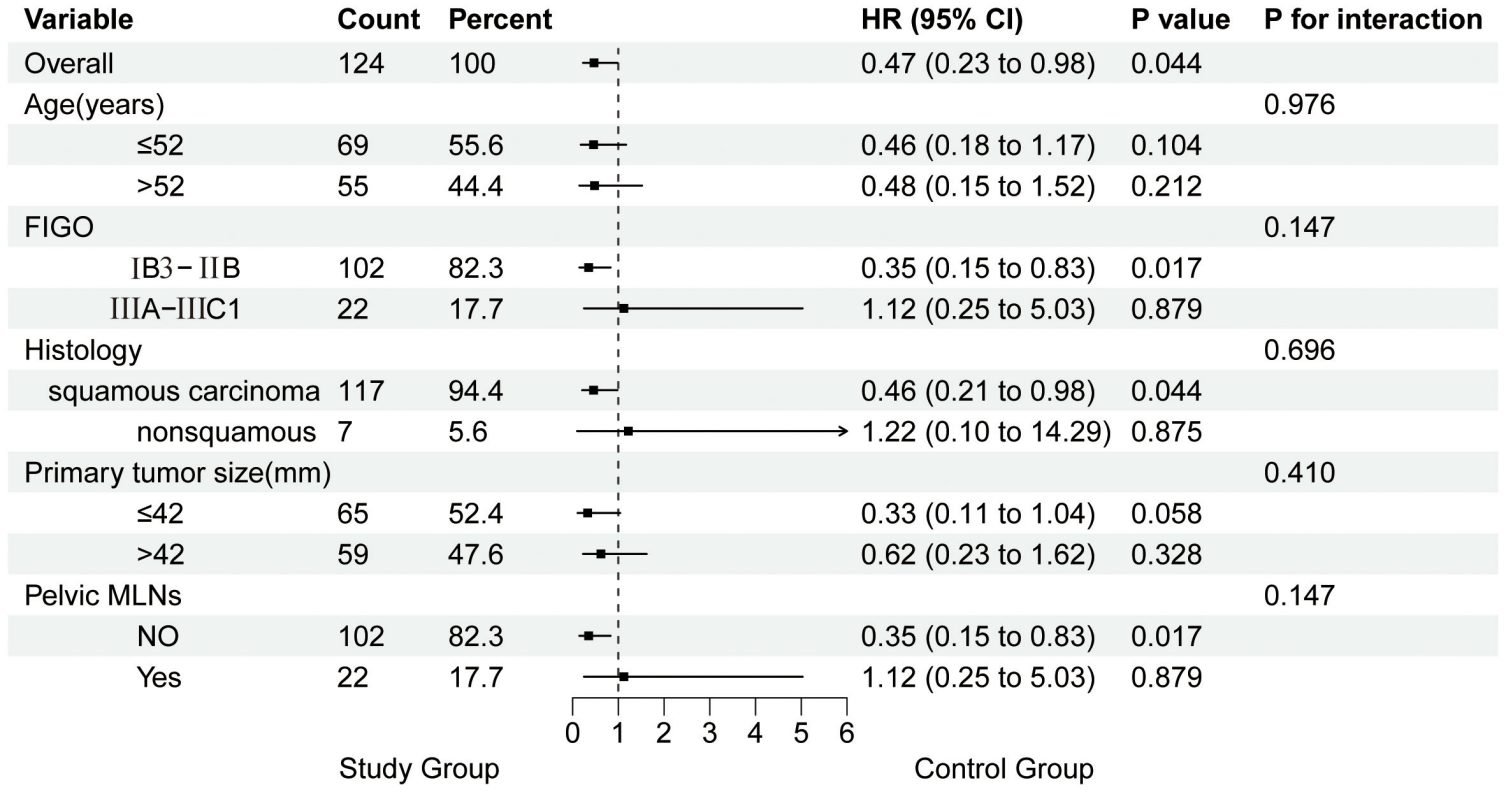
Forest Plot of subgroup analysis and interaction effects on PFS in patients with LACC. MLNs, metastatic lymph nodes.

In the overall study population, the treatment group showed better PFS in LACC patients compared to the control group (HR: 0.47, 95% CI 0.23–0.98, P = 0.044). On subgroup analysis, patients with FIGO stage IB3-IIB, squamous cell carcinoma, and no pelvic lymph node metastasis showed better PFS in the treatment group (P < 0.05). The interaction test results were not statistically significant, indicating that the treatment effects between the study group and the control group were consistent across different subgroups (P for interaction > 0.05). The Forest plot of subgroup analysis and interaction effects on PFS in LACC Patients is presented in **Figure 2**.

### Toxicity and side effects

After PSM, the incidence of grade 2 acute radiation-induced intestinal injury in the study group (1 case [1.6%]) was significantly lower than that in the control group (10 cases [16.1%]). None of the patients in either group experienced grade 3 or higher acute gastrointestinal or urinary toxicity. The study group had 4 cases (6.5%) of grade 3–4 leukopenia and 2 cases (3.2%) of anemia, compared to 18 cases (29.0%) of leukopenia and 14 cases (22.6%) of anemia in the control group, with significantly fewer occurrences in the study group (*P*<0.05). There was no significant between-group difference regarding the incidence of thrombocytopenia (**Table 3**).

**Table 3:** Adverse events in the study group and control group before and after PSM.

	Before PSM (*n*=161)			After PSM (*n*=124)		
	Study Group (*n*=76)	Control Group (*n*=85)	*P* value	Study Group (*n*=62)	Control Group (*n*=62)	*P* Value
Acute radiation-induced bowel injury						
Grade 0–1	75 (98.7)	71 (83.5)	0.001*	61 (98.4)	53 (83.9)	0.004*
Grade 2	1 (1.3)	14 (16.5)		1 (1.6)	10 (16.1)	
Chronic radiation-induced bowel injury^#^						
Grade 0–2	75 (98.7)	81 (95.3)	0.371	61 (98.4)	59 (95.2)	0.309
Grade 3–4	1 (1.3)	4 (4.7)		1 (1.6)	3 (4.8)	
Acute urinary adverse events						
Grade 0–1	73 (96.1)	74 (87.1)	0.043*	59 (95.2)	55 (88.7)	0.187
Grade 2	3 (3.9)	11 (12.9)		3 (4.8)	7 (11.3)	
Chronic urinary adverse events						
Grade 0–2	67 (88.2)	81 (95.3)	0.097	55 (88.7)	59 (95.2)	0.189^#^
Grade 3–4	9 (11.8)	4 (4.7)		7 (11.3)	3 (4.8)	
Leukopenia						
Grade 0–2	69 (90.8)	62 (72.9)	0.004*	58 (93.5)	44 (71.0)	0.001*
Grade 3–4	7 (9.2)	23 (27.1)		4 (6.5)	18 (29.0)	
Thrombocytopenia^#^						
Grade 0–2	75 (98.7)	82 (96.5)	0.623	62 (100)	59 (95.2)	0.122
Grade 3–4	1 (1.3)	3 (3.5)		0 (0)	3 (4.8)	
Anemia						
Grade 0–2	72 (94.7)	70 (82.4)	0.015*	60 (96.8)	48 (77.4)	0.001*
Grade 3–4	4 (5.3)	15 (17.6)		2 (3.2)	14 (22.6)	

Data presented as frequency (percentage). ^#^indicates the use of Fisher’s Exact Test; the Chi-Square Test was applied for the other comparisons. *P<0.05.

PSM, propensity score matching.

## Discussion

Cervical cancer is the fourth most common malignancy among women worldwide, with more than two-thirds of patients presenting with LACC at diagnosis (FIGO 2009/2018: Stage IB2-IVA/IB3-IVA) (1, 10, 18, 19). Previous research indicates that platinum-based CCRT significantly improves OS (HR: 0.68, 95% CI 0.57–0.80) and PFS (HR: 0.63, 95% CI 0.50–0.76) compared to radiotherapy alone (20). However, approximately 50% of all LACC patients treated with CCRT experience disease recurrence or distant metastasis (21, 22), and the associated toxicity severely affects their quality of life. Surgical treatment for LACC carries risks such as extensive trauma, significant bleeding, high postoperative complication rates, pelvic metastasis, and a higher recurrence rate (19). While some reports suggest that preoperative brachytherapy combined with radical surgery achieves favorable clinical outcomes, long-term survival data remain scarce. In this study, we retrospectively compared the survival outcomes and adverse effects of preoperative brachytherapy combined with radical surgery versus CCRT for LACC.

In previous studies, patients with LACC receiving CCRT had 5-year PFS rates ranging from 51% to 80.4% (4, 23, 24) and 5-year OS rates ranging from 55% to 82.5% (4, 23–26). In the present study, the 5-year PFS and OS rates in the CCRT group after PSM were 62.7% and 74.9%, respectively, consistent with prior findings. Moreover, the study group had significantly higher 1-, 3-, and 5-year PFS rates (88.3%, 81.2%, and 81.2%, respectively) compared to the control group (80.6%, 65.2%, and 62.7%, respectively). These results suggest that preoperative brachytherapy combined with radical surgery may offer a survival advantage over CCRT in LACC patients in terms of PFS, although further studies are required to obtain more robust evidence. One potential explanation is that many LACC patients have large primary tumors and a high tumor burden. Thus, CCRT is less likely to eradicate all tumor cells, thereby increasing the risk of recurrence. Additionally, most patients in this study had squamous cell carcinoma, which is more sensitive to radiotherapy (27). Preoperative brachytherapy can substantially downstage tumors, reducing their size, decreasing tumor cell activity, and alleviating parametrial invasion. This enables patients who were initially deemed inoperable to become suitable candidates for radical surgery, thereby eliminating the basis for tumor growth. After PSM, the 5-year OS in the study group (81.4%) was higher than that in the control group (74.9%), although the difference was not statistically significant. This may be due to the small sample size in this single-center, retrospective study.

Multivariate analysis of factors affecting disease progression revealed that preoperative brachytherapy combined with radical surgery was an independent prognostic factor for PFS (HR: 0.458, 95% CI 0.221–0.945, *P*=0.035) after PSM, reducing the risk of disease progression by 54.2% compared to CCRT. This finding further suggests that preoperative brachytherapy combined with radical surgery may help improve the prognosis of LACC patients.

After univariate and multivariate Cox regression analysis, we further performed subgroup analysis and analyzed the interaction effects on PFS in LACC patients. The results of subgroup analysis indicated that patients with FIGO stage IB3-IIB (without pelvic lymph node metastasis) and squamous cell carcinoma were most likely to benefit from preoperative brachytherapy combined with surgery. However, the lack of statistical significance in the interaction test was likely attributable to reduced sample size in each subgroup, leading to inaccurate estimates and wider confidence intervals, affecting the significance of the P-value. Increasing the sample size is expected to enhance the reliability of the results and the statistical power of the analysis.

Adverse effects were a major focus of this study. In the PSM cohort, only 1 patient (1.6%) in the study group developed grade 2 acute radiation-induced bowel injury compared to 10 patients (16.1%) in the control group, indicating that CCRT increases the risk of bowel injury. This is likely because the study group received only three-dimensional (28) intracavitary brachytherapy without EBRT, resulting in lower radiation doses, fewer treatment sessions, and shorter treatment times. Additionally, some patients in the control group with pelvic lymph node metastasis underwent EFRT, which increased radiation exposure to organs at risk, such as the small intestine, potentially leading to a higher incidence of gastrointestinal side effects (29). In addition, the study group had 1 case (1.6%) of partial intestinal obstruction as a short-term surgical complication, and 4 cases (6.5%) of intestinal adhesions as long-term postoperative complications. Bone marrow suppression was a common adverse effect, with significantly fewer cases of grade 3-4 leukopenia (6.5% vs. 29.0%) and anemia (3.2% vs. 22.6%) in the study group compared to the control group. Previous studies (30) have demonstrated that hematologic toxicity is primarily caused by the cytotoxic effects of chemotherapeutic agents (31, 32), and combining chemotherapy with radiotherapy increases the incidence of acute hematologic toxicity by 5%–37%. There was a trend toward a higher incidence of grade 3-4 chronic urinary adverse events in the study group (11.3% vs. 4.8%), although the between-group difference was not statistically significant. This may be due to the use of a type C hysterectomy (Querleu-Morrow classification) (14) in the study group, which involves manipulation of the bladder or ureters and damage to the autonomic nerves, leading to ureteral leakage, urinary incontinence, retention, or difficulty (33). The details of surgical complications are provided in **Supplementary Material**. None of the patients in either group experienced ≥ grade 3 acute gastrointestinal or urinary toxicity, and no treatment-related deaths were observed. Overall, the study group had a better quality of life compared to the control group.

Some limitations of this study should be considered. First, its retrospective design may have introduced an element of bias, despite the use of PSM to balance known variables. Unrecognized or uncollected factors may still affect the conclusions. Secondly, the study’s reliance on hospital-based registry data may lead to selection bias and information bias, potentially resulting in overestimated survival rates. Lastly, this study had a small sample size. Larger, multicenter prospective trials are required to obtain more robust evidence.

In conclusion, in this study, preoperative brachytherapy combined with radical surgery was found to improve PFS in LACC patients, while reducing the incidence of acute radiation-induced bowel injury and hematologic toxicity compared to CCRT. Prospective randomized controlled trials are required to verify our findings.

## Data Availability

The data analyzed in this study is subject to the following licenses/restrictions: the datasets are not included in the article or **Supplementary Material** due to patient confidentiality and privacy concerns. Requests to access these datasets should be directed to Honggui Zhou, hongguizhou@163.com.
