# Supplementary material for: A comparative analysis of survival outcomes and adverse effects between preoperative brachytherapy with radical surgery and concurrent chemoradiotherapy in patients with locally advanced cervical cancer
Source: Front Oncol. 2025 Feb 28;15:1511748. doi: 10.3389/fonc.2025.1511748 (PMC11906330; doi:10.3389/fonc.2025.1511748)
Supplement: Supplementary file 1 [file DataSheet1.docx]

**Treatment Details**

**Treatment Details in the Study Group**

Table 4. Details of preoperative brachytherapy technique

| Technical Parameters | Details |
| --- | --- |
| Brachytherapy machine | MicroSelectron |
| Treatment planning system | ONCENTRA |
| Dose Rate | HDR (High-Dose Rate) |
| Imaging Technology | 3D Technology |
| Radiation Source | 192Ir (Iridium-192) |
| Dose Fractionation | 6Gy per "Point A" dose, 3-4 sessions |
| Total Dose | 18–24 Gy |
| BED (Tumor, α/β = 10) | 28.8–38.4 Gy |
| EQD2 (Tumor, α/β = 10) | 24–32 Gy |

BED, biologically effective Dose; EQD2, equivalent Dose in 2 Gy fractions

**Treatment Details in the Control Group**

The specific details are as follows:

Leukopenia and Thrombocytopenia (Grade 3 or higher): Three patients developed ≥grade 3 leukopenia and thrombocytopenia. One patient was treated with recombinant human granulocyte colony-stimulating factor (150 µg/vial) at a dose of 5 µg/kg (rounded to the nearest vial dose), administered subcutaneously (SC) once daily until the leukocyte count reached ≥10×10^9^/L and the platelet count reached ≥100×10^9^/L. Another patient developed grade 4 thrombocytopenia and was treated with recombinant human thrombopoietin (rhTPO, 300 U/kg), administered subcutaneously once daily until the platelet count increased by 50×10^9^/L above baseline, at which point the medication was discontinued. Treatment duration was extended by 7-14 days. One more patient developed ≥grade 3 leukopenia and thrombocytopenia after 8 weeks of treatment and received the same treatment.

Leukopenia and Anemia (Grade 3 or higher): Six patients developed ≥grade 3 leukopenia and anemia. One patient was treated with recombinant human granulocyte colony-stimulating factor (150 µg/vial) at a dose of 5 µg/kg (rounded to the nearest vial dose), administered subcutaneously once daily. Erythropoietin (EPO, 3000 U, three times weekly) and oral iron supplements (Polysaccharide iron complex capsule (150 mg), administered orally once daily) were given until hemoglobin reached ≥120 g/L. Treatment was extended by 7 days. Five patients developed grade 3 or higher leukopenia and anemia after completing the full 8-week treatment course and were treated similarly.

Leukopenia (Grade 3 or higher): Nine patients developed ≥grade 3 leukopenia, with seven receiving recombinant human granulocyte colony-stimulating factor (150 µg/vial) at a dose of 5 µg/kg (rounded to the nearest vial dose), administered subcutaneously once daily. The treatment duration was extended by 3-7 days. The remaining two cases occurred after the full 8-week treatment course.

Anemia (Grade 3 or higher): Eight patients developed ≥grade 3 anemia after 8 weeks of treatment. These patients were treated with erythropoietin (EPO, 3000 U, three times weekly) and iron supplements (Polysaccharide iron complex capsule (150 mg), administered orally once daily) until hemoglobin reached ≥120 g/L.

Acute Radiation-Induced Enteritis (Grade 2): Ten patients developed grade 2 acute radiation-induced enteritis, three of whom were primarily treated with Cefaclor sustained-release capsules (375 mg), administered orally twice daily; Levofloxacin tablets (500 mg), administered orally once daily; Yunnan Baiyao (0.5 g), administered orally four times daily, for symptomatic treatment, with treatment duration extended by 5-7 days. The remaining seven cases occurred after 8 weeks.

Acute Radiation-Induced Cystitis (Grade 2): Seven patients developed grade 2 acute radiation-induced cystitis, three of whom were treated with tranexamic acid (1.0 g), administered intravenously once daily; Cefaclor sustained-release capsules (375 mg), administered orally twice daily; levofloxacin tablets (500 mg), administered orally once daily; and were advised to increase fluid intake. The remaining four cases occurred after 8 weeks.

**The OR values for PFS and OS**

The odds ratios (OR) for progression-free survival (PFS) and overall survival (OS) calculated using contingency tables, are as follows:

PFS:
In the study group, 11 patients had disease progression while 51 had no progression. In the control group, 22 patients showed disease progression while 40 showed no progression. The OR was calculated as follows:
OR = (11 × 40) / (22 × 51) = 440 / 1122 ≈ 0.392, 95% CI [0.170, 0.903], P = 0.025.
This indicates that the odds of progression in the study group were 39.2% of those in the control group, representing a significantly lower risk of progression. The 95% confidence interval (CI) does not include 1, confirming the statistical significance of this result.

OS:
In the study group, 10 patients died, and 52 survived. In the control group, 13 patients died, and 49 survived. The OR was calculated as follows:
OR = (10 × 49) / (13 × 52) = 490 / 676 ≈ 0.725, 95% CI [0.291, 1.805], P = 0.488.
Although the study group had a lower mortality rate than the control group, this difference was not statistically significant. The 95% CI includes 1, indicating insufficient evidence to confirm a significant difference in mortality risk between the two groups.

**Surgical complications**

Short-term complications:
Wound healing complications: Two patients experienced poor wound healing, but in both cases, the infection resolved with dressing changes and wound care.
Ureteral leakage: One patient developed ureteral leakage, which was successfully treated with a subsequent bladder-ureteral anastomosis.
Urinary retention: Five patients experienced urinary retention. All cases were managed by extending the indwelling catheter placement, with successful catheter removal within 3-4 weeks.
Intestinal obstruction: One patient experienced partial intestinal obstruction, which was resolved with gastrointestinal decompression and fasting.

Long-term complications:
Bowel adhesion: Four patients developed bowel adhesions, presenting with mild symptoms such as abdominal pain and constipation. These symptoms were self-limiting and resolved without additional intervention.
